# 3M-CPSEED, An EEG-based Dataset for Chinese Pinyin Production in Overt, Mouthed, and Imagined Speech

**DOI:** 10.1038/s41597-025-06346-1

**Published:** 2025-12-08

**Authors:** Xinyu Ma, Yi Jiang, Ning Jiang

**Affiliations:** 1 National Clinical Research Center for Geriatrics, West China Hospital Sichuan University, Chengdu, Sichuan Province China; 2 Med-X Center for Manufacturing, Sichuan University, Chengdu, Sichuan Province China

**Keywords:** Language, Nanoscience and technology

## Abstract

Speech brain-computer interfaces (BCIs) enable communication with the external world by decoding neural signals. However, language function as a higher-order brain function, the neural mechanisms underlying speech production remain incompletely understood. Currently most existing Chinese EEG datasets use sentences as stimuli, overlooking that Pinyin constitutes the phonetic foundation of Chinese characters, which limits research on decoding individual Chinese character components. Moreover, most datasets employ only one speech production paradigm, preventing exploration of the brain’s diverse speech production modes. This study aims to construct the 3M-CPSEED Chinese Pinyin dataset for exploring neural activity during three distinct speech modes (overt speech, silently articulated speech, imagined speech)of syllables from distinct articulatory positions. The dataset comprises EEG recordings from 20 participants completing four experimental blocks within one day, yielding 1,800 validated trials. 3M-CPSEED holds significant implications for speech neurophysiology research, not only facilitating exploration of neural activity differences across pinyin articulations but also enabling robust transfer learning studies for other alphabetic languages.

## Background & Summary

As a high-level brain function, speech processing enables rapid comprehension and production of linguistic information, which exemplifies the brain’s advanced neural computational abilities^1^. Generally speaking, damage to specific brain regions caused by stroke, brain injury, tumors, or neurodegeneration can trigger aphasia^2^. The impairment of language function will significantly reduce the quality of life of patients, which may lead to depression, anxiety and other mental disorders in severe cases. Currently, aphasia rehabilitation remains a major clinical challenge. As a potential technology, brain computer interface (BCI) can establish a direct communication path between the brain and external devices by decoding the information of electrophysiological signals of the central nervous system^3,4^. Specifically, it directly converts the user’s “internal voice” intention into an outputable communication signal by decoding the neural activities induced by the speech imagery process in real time, thus providing an opportunity for patients who have lost basic language communication ability to restore communication, improve the quality of life and maintain independence^5–9^. However, at present, the precise brain regions involved in language function are not fully understood, and the related neural mechanisms are still under exploration. Therefore, it is necessary to further study the neural signals in the process of speech imagery based on BCI technology, which provides a key way to further explore the neural representation related to language production. More importantly, an in-depth understanding of these neural representations also provides a prerequisite for the development of high-performance speech imaging based BCI systems.

Currently, multiple neural signal modalities are employed for imagined speech decoding, including electroencephalography (EEG), functional magnetic resonance imaging (fMRI), and electrocorticography (ECoG)^10–15^. Among these, EEG is widely used for large-scale data studies due to its non-invasiveness, cost-effectiveness, and high temporal resolution^16^. Within the field of imagined speech decoding, the majority of invasive studies require electrode implantation in speech-related cortical areas to restore partial language function^17–19^. However, the surgical risks limit its wider adoption in healthy individuals or non-critical cases. In recent years, non-invasive BCI research utilizing naturalistic paradigms has grown significantly^20^. In these paradigms, participants are exposed to stimuli resembling real-world experiences, and their EEG signals during this process are decoded. Compared to traditional paradigms relying on meticulously designed stimuli (e.g., P300, visual evoked potentials, slow cortical potentials), naturalistic paradigms offer higher ecological validity and user acceptance. Several public datasets, such as ZuCo^21^, ChineseEEG^22^, BCCWJ-EEG^23^, and Hsg^24^, employ sentence-level stimuli, offering valuable resources to validate non-invasive BCIs in imagined speech applications.

At present, Chinese is the most spoken native language in the world. However, the limitations in linguistic characteristics and phonetic unit granularity severely restrict the development of Chinese-language BCIs. Chinese is a typical tonal language. Previous studies primarily focused on non-tonal languages like English^18,19,25–29^, Spanish^30^, and Dutch^31–34^, yet neural mechanisms for processing different languages vary significantly. For example, Chinese engages distinct neural circuits and representation patterns compared to English, particularly in brain regions related to tone perception and production^35^. This language specificity impedes direct application of neural decoding models and feature extraction methods developed for non-tonal languages to Chinese. Although a few public datasets include Chinese imagined speech, they primarily target sentence-level decoding^22,36–38^ and lack datasets focusing on Pinyin—the core phonetic unit underlying Chinese characters. This gap leads to two critical limitations: 1) It hinders investigation into neural representations of Chinese phonetic elements (initials, finals)—essential for understanding character processing and building efficient phoneme-level decoders; 2) While alphabetic systems (e.g., English/German/Spanish) use phoneme combinations, Chinese Pinyin’s standardized phonemic annotation could enable cross-linguistic comparisons of neural encoding for phoneme-level processing (e.g., consonant/vowel commonalities). However, the absence of high-quality imagined speech datasets specifically designed for Chinese Pinyin prevents such cross-linguistic studies, limiting universal advancements in speech BCI technology. Thus, developing a dataset based on Pinyin, the fundamental phonetic unit of Chinese, is crucial.

Beyond language type, another key dimension for characterizing speech imagery tasks involves the modality of speech production. The primary modalities include overt speech, silently articulated speech, and imagined speech. Overt speech involves full articulator movement with vocal output. Silently articulated speech maintains articulator motion (e.g., lips/tongue/jaw) without sound, retaining strong motor commands and proprioceptive feedback. Imagined speech internally simulates speech without measurable movement or sound, relying primarily on high-level cognition and motor planning. At present, the “Imagined speech” paradigm has become the mainstream paradigm of current research due to its low external interference and potential BCI practicability^36,37^. Existing public datasets typically focus on a single paradigm and lack datasets for the three modalities of “overt speech”, “silently articulated speech” and “imagined speech” within the same sample.This gap prevents systematic comparisons of neural dynamics across modalities, limiting exploration of speech production mechanisms and applications in transfer learning^39^. Furthermore, because overt and silently articulated speech involve coordinated articulator movements (e.g., lips, tongue), analyzing EEG signals from Pinyin with distinct articulatory positions during these modalities could reveal fine-grained encoding patterns in motor cortex (e.g., primary motor cortex M1, supplementary motor area SMA) and sensorimotor integration regions. Specifically, this approach may uncover hierarchical organization and articulation-specific responses at the monosyllable level. Thus, datasets integrating both articulatory positions and production modalities are critical for deciphering the neural mechanisms underlying speech production.

We introduce the 3M-CPSEED EEG dataset of Chinese Pinyin to address the research gap in Pinyin-level neural representations and investigate parallels and distinctions in neurodynamics across three speech production modes along with their dependencies on articulation positions. We recorded EEG data from 20 healthy native Chinese speakers across the four experimental blocks within a single day, totaling approximately 1.5 hours. Participants performed tasks using 10 representative Pinyin (a, i, u, ü, m, f, j, l, k, ch) covering major articulatory positions, following specific instructions for overt speech, silently articulated speech, and imagined speech paradigms. This design generated 450*4 = 1800 valid trials (3*3*50 = 450 trials per paradigm, 5*9 = 45 repetitions per Pinyin in each paradigm). The dataset provides raw EEG data, preprocessed signals, and corresponding labels, enabling researchers with limited cross-disciplinary expertise to conduct immediate analysis. As the first public EEG resource exclusively focusing on Chinese Pinyin with synchronous three-paradigm comparison, 3M-CPSEED: 1. Enables deep investigation of neural representations for Chinese phonetic elements (initials, finals) across different mode; 2. Reveals gradient changes in motor cortex activity patterns during encoding of distinct articulatory positions across task paradigms, clarifying neurodynamic processes in speech motor planning, execution, and suppression; 3. Facilitates developing optimized decoding models for syllable-level Chinese speech BCIs; 4. Advances cross-linguistic studies on neural encoding universality and specificity through comparison with phoneme-based datasets (e.g., English).

## Methods

### Participants

All participants provided written informed consent before the experiment. The experimental protocol was approved by the Ethics Committee of West China Hospital (Approval No.: 2025(770)) and conformed to the Declaration of Helsinki (2013)^40^. Twenty healthy native Mandarin speakers participants (mean ± SD: 24.55 ± 2.58 years) including 11 females and 9 males were enrolled. All participants were right-handed with normal or corrected-to-normal vision, normal hearing, and no history of speech, neurological, motor, or psychiatric disorders. Each participant completed four blocks, with each block comprising 600 trials per speech mode (overt speech, silently articulated speech, imagined Speech). The total experimental duration per participant was approximately 1.5 hours. Participants were labeled as Sub 1 to Sub 20, with detailed demographics summarized in Table 1.

**Table 1:** Participants information.

Session	Gender	Age
Sub1	Male	25
Sub2	Female	25
Sub3	Male	29
Sub4	Male	27
Sub5	Male	23
Sub6	Female	25
Sub7	Female	30
Sub8	Female	26
Sub9	Male	21
Sub10	Female	23
Sub11	Female	24
Sub12	Female	21
Sub13	Male	27
Sub14	Female	25
Sub15	Male	24
Sub16	Female	25
Sub17	Male	24
Sub18	Female	19
Sub19	Female	24
Sub20	Male	24

### Materials

As the foundational component of Chinese characters, Pinyin constitutes the initial learning step for all native Chinese speakers. Decoding Pinyin effectively facilitates Chinese character recognition. Therefore, our stimulus materials were presented in the form of individual Pinyin syllables. Chinese Pinyin comprises Finals and Initials, which are further classified according to distinct articulation manners and positions, as detailed in Tables 2 and 3. Given the fundamental differences between Initials and Finals in syllabic structure and articulatory physiology, and to further investigate the influence of different manners of articulation and places of articulation on speech production, this study carefully constructed a highly representative and contrasting set of Pinyin stimuli: Finals: “a, i, u, ü”; Initials: “m, f, j, l, k, ch”. Additionally, to avoid potential confusion from tonal variations, all Pinyin stimuli were pronounced using a flat tone during the experiment.

**Table 2:** Categorization of Chinese Finals by Articulatory Configuration.

	Single Finals
Open mouth	**a**, o, e
Spread lip	**i**
Rounded lip	**u**
Lip protrusion	**ü**

**Table 3:** Categorization of Chinese Initials by Articulatory Configuration.

	Bilabial	Labiodental	Alveolar	Alveolo-Palatal	Retroflex	Velare
Plosive	b, p		d, t			g, **k**
Fricative		**f**	s	x	sh, r	h
Affricate			z, c	**j**, q	zh, **ch**	
Nasal	**m**		n			
Lateral			**l**			

To strike a balance between comprehensively capturing the articulatory features of the Chinese phonological system and maintaining a concise, controllable set of stimuli, we selected this set of Pinyin sounds. Key distinguishing dimensions for simple finals include: vowel height (high/low), frontness/backness, roundedness, and specific mouth shape during articulation. “a” was selected as the sole low final with an unrounded, open-mouth articulation. “i” is the only high front unrounded final with a spread-lip articulation. “u” is the only high back rounded final with rounded-lip articulation. “ü“is a distinctive Chinese close front rounded vowel with lip protrusion. Initials are primarily distinguished by place of articulation (Bilabial, Labiodental, Alveolar, Alveolo-Palatal, Retroflex, Velare) and manner of articulation (Nasal, Fricative, Affricate, Lateral, Plosive). Due to the large number of initial consonants, choosing fewer initial consonants to form our dataset while covering all vocalization characteristics can effectively shorten the experimental time and ensure the number of experiments, among other benefits. “m” was chosen as a bilabial nasal. “f” was selected as a labiodental fricative. “j” was chosen as a palatal affricate. “l” was selected as an alveolar lateral. “k” was chosen as a velar plosive. “ch” was selected as a retroflex affricate^41^. These initials exhibit distinct physiological differences in articulation, enabling effective investigation of how different speech mechanisms influence brain activation patterns. Furthermore, since the articulation of Initials relies on their combination with Finals, we employed canonical pairings from Chinese language teaching practice, as detailed in Table 4. All pronunciations were standardized through participant training during the preparatory phase prior to the experiment.

**Table 4:** Initial consonants and their corresponding vowels.

	Bilabial	Labiodental	Alveolar	Alveolo-Palatal	Retroflex	Velare
Plosive	/bo/, /po/		/de/, /te/			/ge/, /ke/
Fricative		/fo/	/si/	/xi/	/shi/, /ri/	/he/
Affricate			/zi/, /ci/	/ji/, /qi/	/zhi/, /chi/	
Nasal	/mo/		/ne/			
Lateral			/le/			

### Data acquisition

**Fig. 1:**
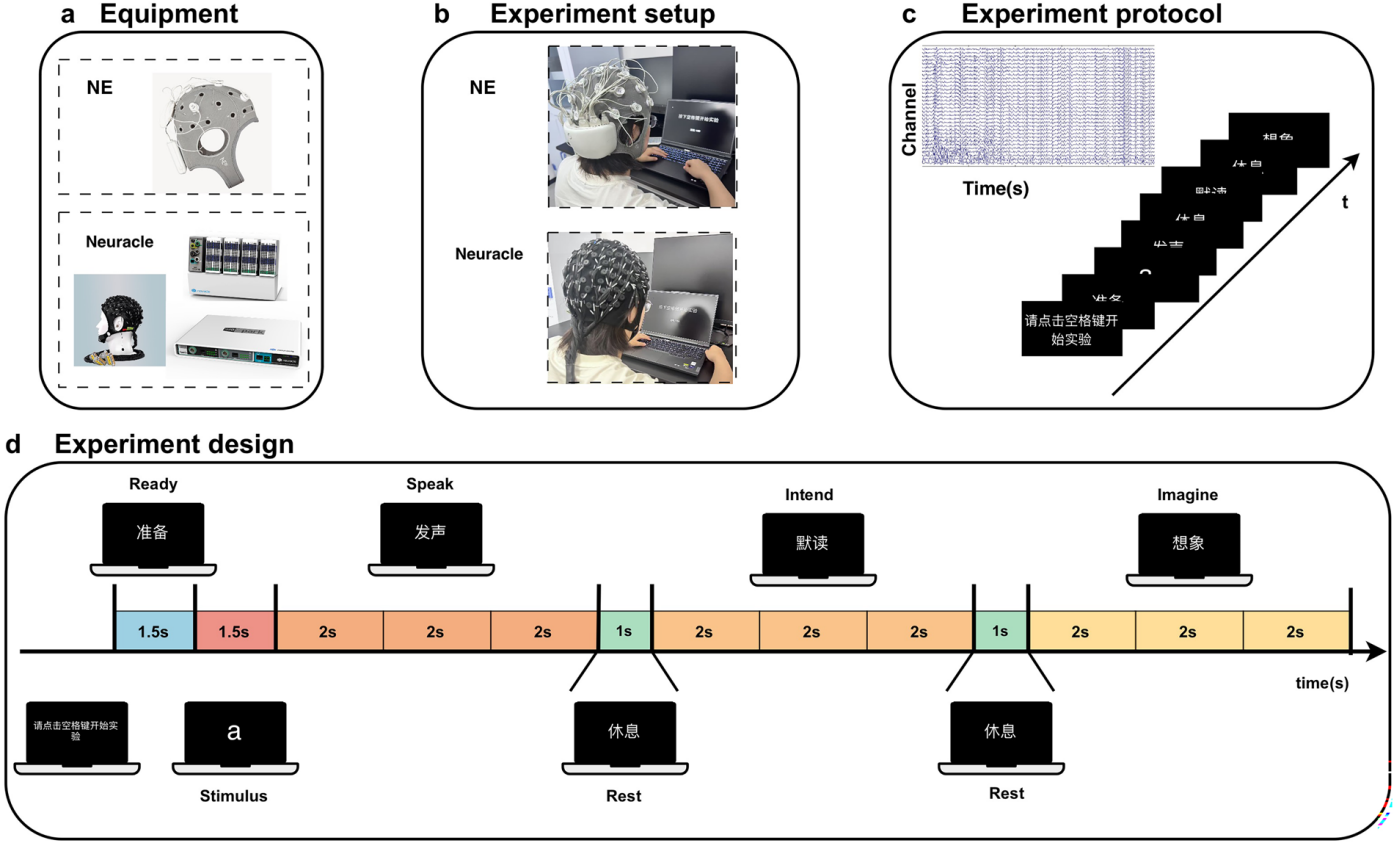
Overview of the experiment. (**a**) Equipment. EEG caps and amplifiers from NE and Neuracle were employed. (**b**) Setup. Participants sat quietly 70 cm from a 24-inch monitor, executing tasks per on-screen instructions. (**c**) Protocol. EEG signals were recorded during three speech phases: overt, silently articulated, and imagined speech of Pinyin syllables. (**d**) Block structure. Each block comprised 50 self-initiated trials; the schematic details a single trial’s 23 s sequence.

This study was conducted in an electrically shielded room. Participants were seated in a chair positioned 70 cm from the screen, facing its center directly. To familiarize participants with the environment and procedures, we first explained the informed consent document and outlined the experimental protocol. Then an EEG cap with electrodes was fitted; this preparation phase lasted approximately 50 minutes. Figure 1 showed the main experimental design.

Data from Sub 1 to 15 were collected using a 32-channel NE Enobio EEG system. We placed the electrodes according to the international 10–20 system, with each electrode being circular Ag/AgCl with a diameter of 12 mm. For Sub 16 to 20, we employed a Neuracle NeuSen H 128-channel EEG device. Electrode placement followed the international 10–10 system, and each electrode was Ag/AgCl with a total contact area of 87 mm². Neither system incorporated built-in filters. Since the 32-channel layout (10–20 system) is a standard subset of the 128-channel layout (10–10 system), the data from the corresponding channels in the 128-channel recordings could be directly extracted without spatial interpolation. Meanwhile, the caps of both EEG brands were of the same size, M (54–58 cm). Impedance was checked before each experimental session and maintained below 30 kΩ. Stimuli were presented on a 13.6-inch computer monitor with a refresh rate of 60 Hz and a resolution of 2560 × 1664 pixels. The stimuli were displayed at the center of the screen. To avoid visual fatigue, stimuli were displayed in white on a black background. Participants were encouraged to take breaks during the session. A trigger event marker was set at the onset of each trial to record its precise start time.

Raw EEG data acquired with the NE system were saved in EASY format at a sampling rate of 500 Hz. Raw EEG data acquired with the Neuracle system were saved in folder-based file structures at a sampling rate of 1000 Hz. Stimulus presentation was controlled using Python code. Additionally, to protect participant privacy, no audio or video was recorded during the experiment.

### Experimental design

To avoid eye fatigue, the experimental design employed a black background with white text. Participants were instructed to remain still and articulate the displayed Pinyin at a steady pace throughout the trial. Figure 1(d)details the composition of each trial in the three-phase experiment, including phase durations and cumulative timing. Each trial began with a “Preparation” phase. This was followed by the “Stimulus” phase, where a random Pinyin syllable appeared on screen, which participants were required to memorize. A 1 s “Rest” interval separated the “Stimulus”, “Speak”, “Silently articulated”, and “Imagined” phases. The “Speak” phase lasted 6 seconds total, divided into three 2 s sub-phases. During this phase, participants were required to clearly articulate the displayed Pinyin syllable continuously. The “Silently articulated” phase also lasted 6 s total, divided into three 2 s sub-phases. Participants performed continuous silent articulation at a steady pace, engaging normal tongue and lip movements without producing audible sound. The “Imagined” phase lasted 6 s total, similarly divided into three 2 s sub-phases. During this phase, participants engaged in continuous, steady-paced imagined articulation of the syllable. Each trial was initiated by a manual button press from the participant. This was implemented to mitigate attention lapses due to prolonged repetitive tasks, thereby enhancing data accuracy and reliability.

In the experiment, each of the ten Pinyin syllables appeared five times within a single block. Stimuli were presented in random order to prevent cognitive biases that might arise from fixed repetition sequences. Pinyin syllable in any one of the three tasks (overt speech, silent reading, or imagery) as one trial. Each session consisted of 50 blocks. Participants manually pressed the ‘space’ to initiate each subsequent block. The entire experiment consisted of 50*4 = 200 blocks. Each task phase within a block was divided into three trails. Therefore, each participant completed 200*3 = 600 trials respectively during the ‘Speak’, ‘Intend’, and ‘imagine’ phases, total 600*3 = 1800trails.

To ensure adequate per-syllable sampling ( ≥50 trials) while minimizing total session duration and sustaining high attention engagement, we conducted preliminary testing prior to formal experiments. Preliminary results indicated that a conventional design (recording only one data epoch per stimulus presentation) would exceed two hours to achieve 50 trials per syllable, significantly increasing risks of participant fatigue and attention lapses that compromise data quality. Consequently, we developed an innovative “triple-phase paradigm” to maintain sufficient trial counts while reducing time demands. This ensured participants could complete the experiment while maintaining focused attention. In this paradigm, each 6 s Speak/Intend/Imagine phase following stimulus presentation was segmented into three 2 s data epochs. This design significantly improved data acquisition efficiency, enabling more data collection within participants’ sustained attention span.

### Data Preprocessing

**Fig. 2:**
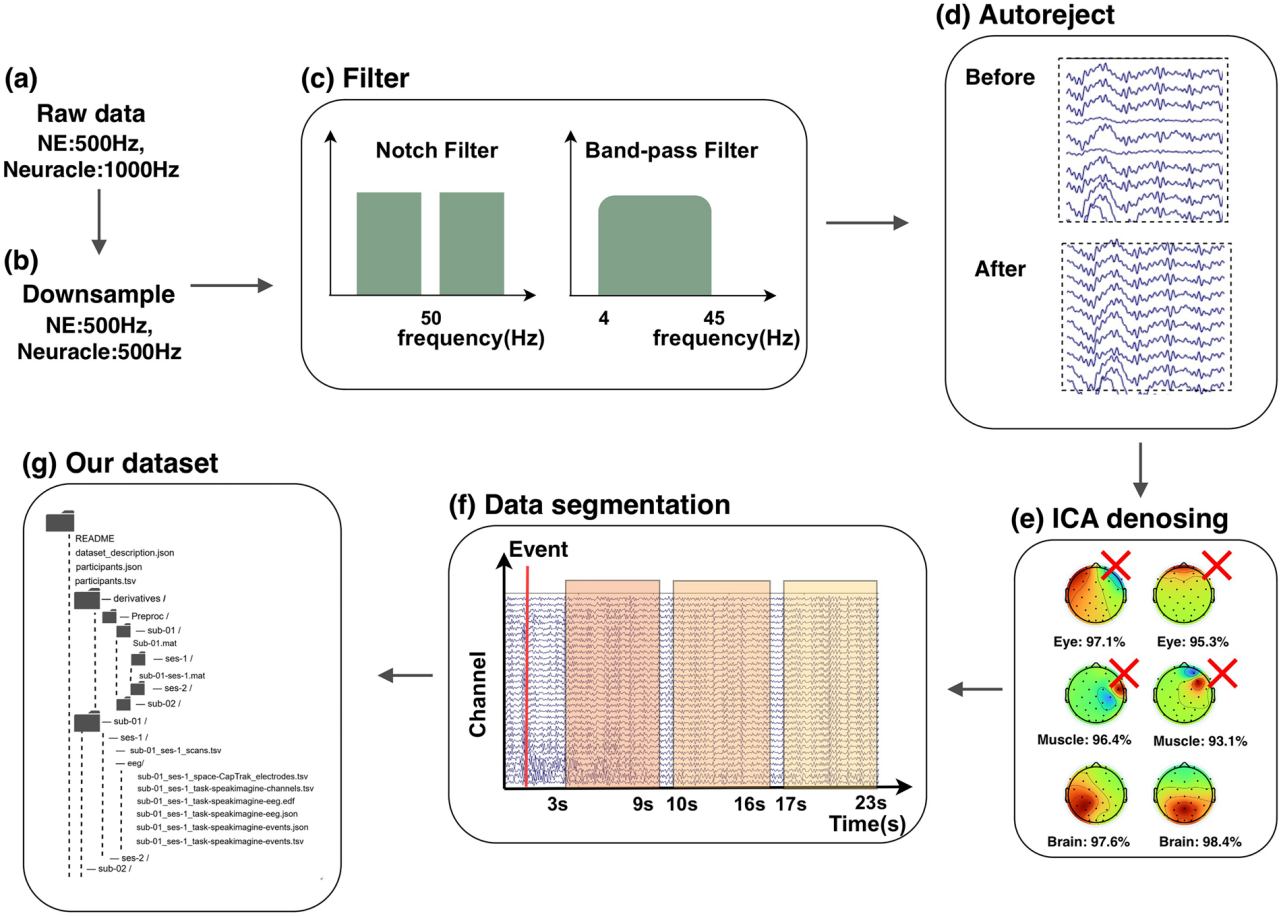
EEG preprocessing pipeline. (**a**) Raw data. NE system recordings (500 Hz;.easy format) and Neuracle system recordings (1000 Hz; folder-structured). (**b**) Downsampling. Neuracle data downsampled to 500 Hz. (**c**) Filtering. Notch filtering (50 Hz removal) and bandpass filtering (4–45 Hz). (**d**) Autoreject. Automated bad channel interpolation. (**e**) ICA denoising. EOG and EMG removal via independent component analysis. (**f**) Data Segmentation. Epoch extraction locked to experimental events. (**g**) Dataset structure. BIDS-formatted organization.

Data preprocessing was implemented in MATLAB, primarily utilizing the EEGLab toolbox^42^. We applied minimal preprocessing to preserve maximal feature integrity for researcher-defined downstream analyses. Additionally, raw data were provided to facilitate custom preprocessing for specific research objectives. The preprocessing pipeline is shown in Fig. 2, mainly including downsampling, filtering, ICA denoising, and data segmentation. The detailed explanations of each step were as follows:

#### Downsampling

To harmonize sampling rates across devices, Neuracle 128-channel data were downsampled to 500 Hz. This retained higher-frequency information while ensuring cross-system comparability.

#### Filtering

A 50 Hz notch filter removed line noise interference. Bandpass filtering (4–45 Hz) was then applied to capture theta (4–8 Hz), alpha (8–13 Hz), beta (13–30 Hz), and low-gamma (30–45 Hz) oscillations.

#### Autoreject

Employing a mean-based algorithm, this approach first computed the time-locked average signal across the entire dataset. From this reference, dynamic channel-specific amplitude thresholds (mean ± 3 SD) were derived. A sliding 50 ms analysis window identified anomalous segments exceeding these thresholds. The pipeline prioritized bad channel interpolation, while discarding entire epochs only when continuous artifacts persisted beyond 200 ms on individual channels. It is noteworthy that this autoreject procedure was applied specifically to data from Subject 19, as this participant’s smaller head circumference resulted in intermittent poor contact for certain channels. For all other participants, standard preprocessing with ICA proved sufficient to achieve high-quality data without the need for additional trial rejection.

#### ICA Denoising

Independent Component Analysis (ICA) is a widely used method for denoising EEG data. This standard technique separates unwanted artifacts from EEG signals through blind source separation. Extended Infomax ICA decomposition was performed. Components classified by EEGLab’s automatic labeling with >90% probability as ocular, muscular, or channel noise artifacts were rejected. This automated pipeline ensured standardized denoising without manual intervention.

#### Data segmentation

Continuous data were segmented around pre-defined event markers, with epoch windows aligned to experimental phases: 1. ‘Speak’ period: [3–5 s], [5–7 s], [7–9 s]. 2. ‘Silently articulated’ period: [10–12 s], [12–14 s], [14–16 s] 3. ‘Imagine’ period: [17–19 s], [19–21 s], [21–23 s].

## Data Records

The EEG data along with basic personal information (age, gender, hand) are publicly accessible. The full dataset, including the raw and preprocessed EEG data, is publicly accessible via the Openneuro platform (https://openneuro.org/datasets/ds006465/versions/2.0.0)^43^. Public data is distributed under the Creative Commons Attribution 4.0 International Public License (https://creativecommons.org/licenses/by/4.0/).

### Data privacy

All data were anonymized by removing potentially identifiable information to protect participant confidentiality. As part of the informed consent procedure, participants were explicitly informed about public data sharing and provided consent for their anonymized data to be openly shared.

### Data Structure

**Fig. 3:**
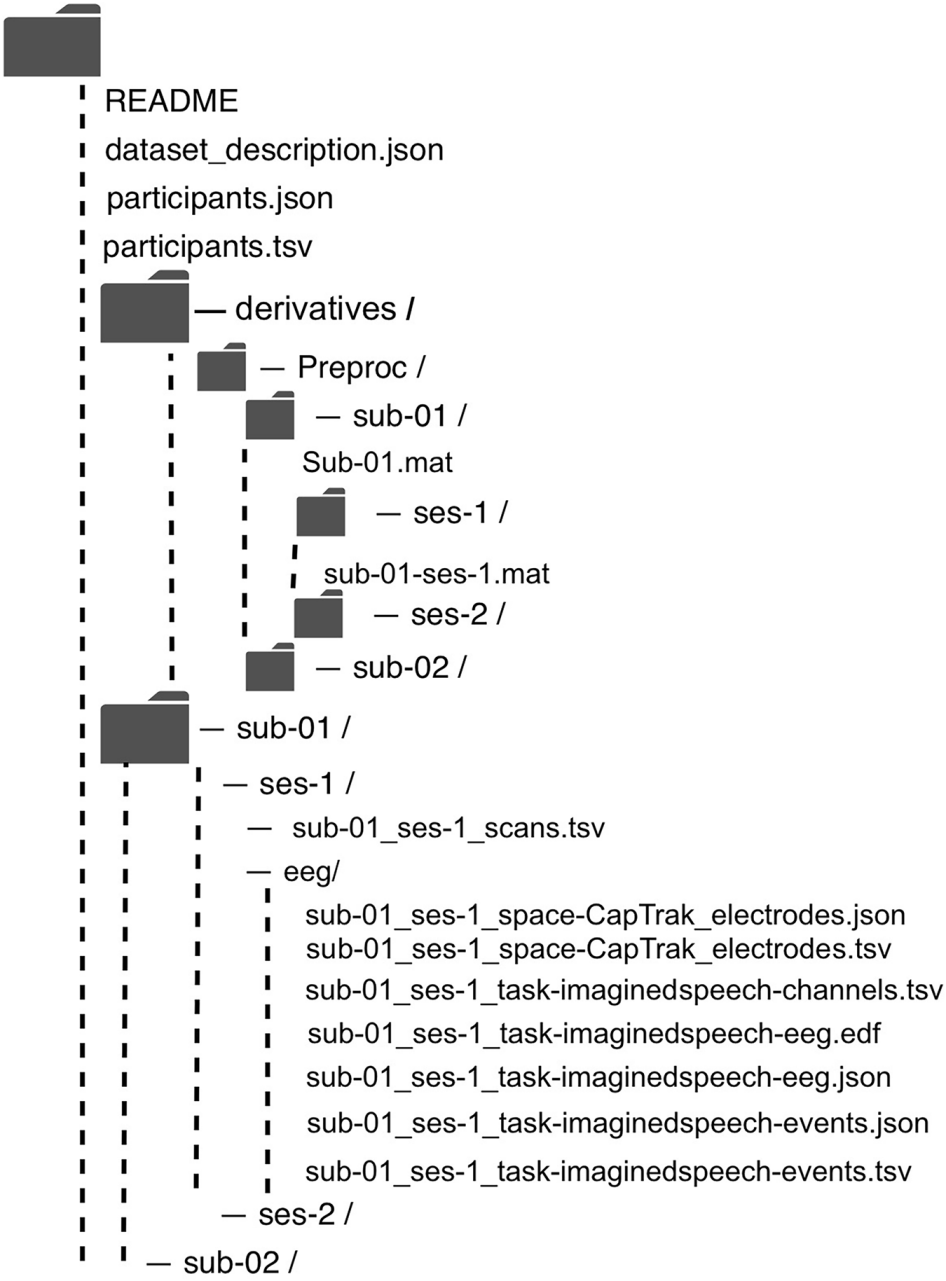
Data Structure.

Raw and preprocessed data are stored in separate directories, with each subject having a dedicated folder containing both data files and corresponding labels. Beyond subject-level data, the main directory also includes: (i) data-description.json: Provides detailed dataset metadata including spatial/temporal parameters. (ii) participants.tsv: Contains participant demographics (gender, age, hand). (iii) participants.json: Documents column-specific metadata for participants.tsv. (iv) README: Contains dataset overview and contact information. Preprocessed data for each subject are provided in the ‘derivatives/preproc’ directory, while raw data are stored in the main directory. The raw data for each subject includes: (i) channels.tsv: Contains number and names of channels; (ii) electrodes.tsv: Contains electrode placement positions of the device used for the subject; (iii) electrodes.json: Documents column-specific metadata for electrodes.tsv; (iv) events.tsv: Contains event information for the current session; (v) events.json: Documents column-specific metadata for events.tsv; (vi) eeg.edf: Contains the raw EEG data for the current session; (vii) eeg.json: Documents detailed information for the EEG data. The file structure is illustrated in Fig. 3.

## Technical Validation

### Classic sensor-level EEG analysis

The EEG data in this dataset enables time-frequency analysis. In this section, we extracted neural oscillations across frequency bands from preprocessed EEG data. Using 0.5–80 Hz bandpass-filtered preprocessed data from Subject 1, we identified time-locked segments corresponding to Imagined Speech trials. Neural activity was analyzed at the C3 and FC5 channels overlying the temporal lobe and Broca’s area respectively, two regions critically involved in speech production.

**Fig. 4:**
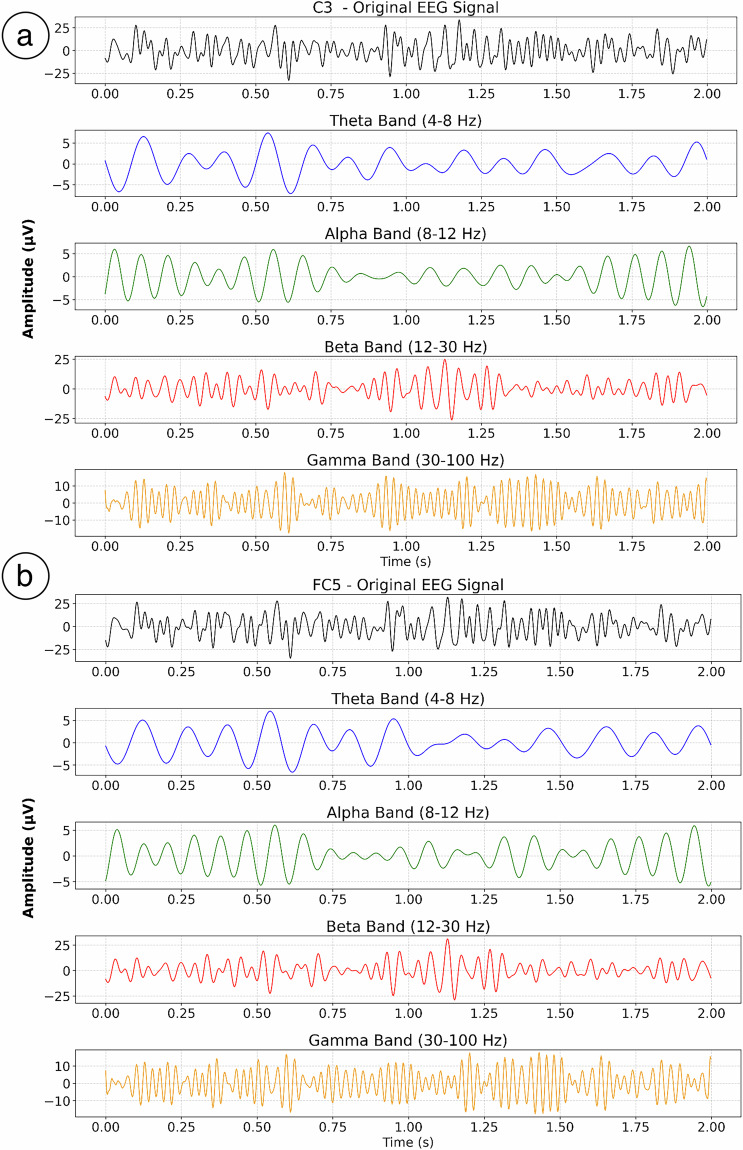
Time-frequency representations and oscillatory activity across frequency bands for Subject 1 during imagined speech trials (4–45 Hz bandpass-filtered). Signals from C3 and FC5 electrodes overlying the temporal cortex and Broca’s area were analyzed.

To isolate intrinsic frequencies in the C3 and FC5 channels, we applied Fast Fourier Transform (FFT) to convert time-domain signals into frequency-domain representations, thereby revealing spectral characteristics of neural activity. Four frequency bands of interest were defined according to standard EEG spectral ranges: Theta (4–8 Hz), Alpha (8–12 Hz), Beta (12–30 Hz), and Gamma (30–45 Hz), enabling classification of neural oscillations by distinct frequency ranges. For each frequency band, the separated components underwent inverse FFT (iFFT) processing to reconstruct time-domain signals. This procedure quantitatively analyzed oscillation amplitudes within each band, confirming that electrophysiological brain activity exhibits distinct oscillatory rhythms, while enabling deeper investigation of neurophysiological processes in specific frequency ranges. Frequency-band-specific results for the C3 and FC5 channels are presented in Fig. 4.

### Channel rejection Rate

**Fig. 5:**
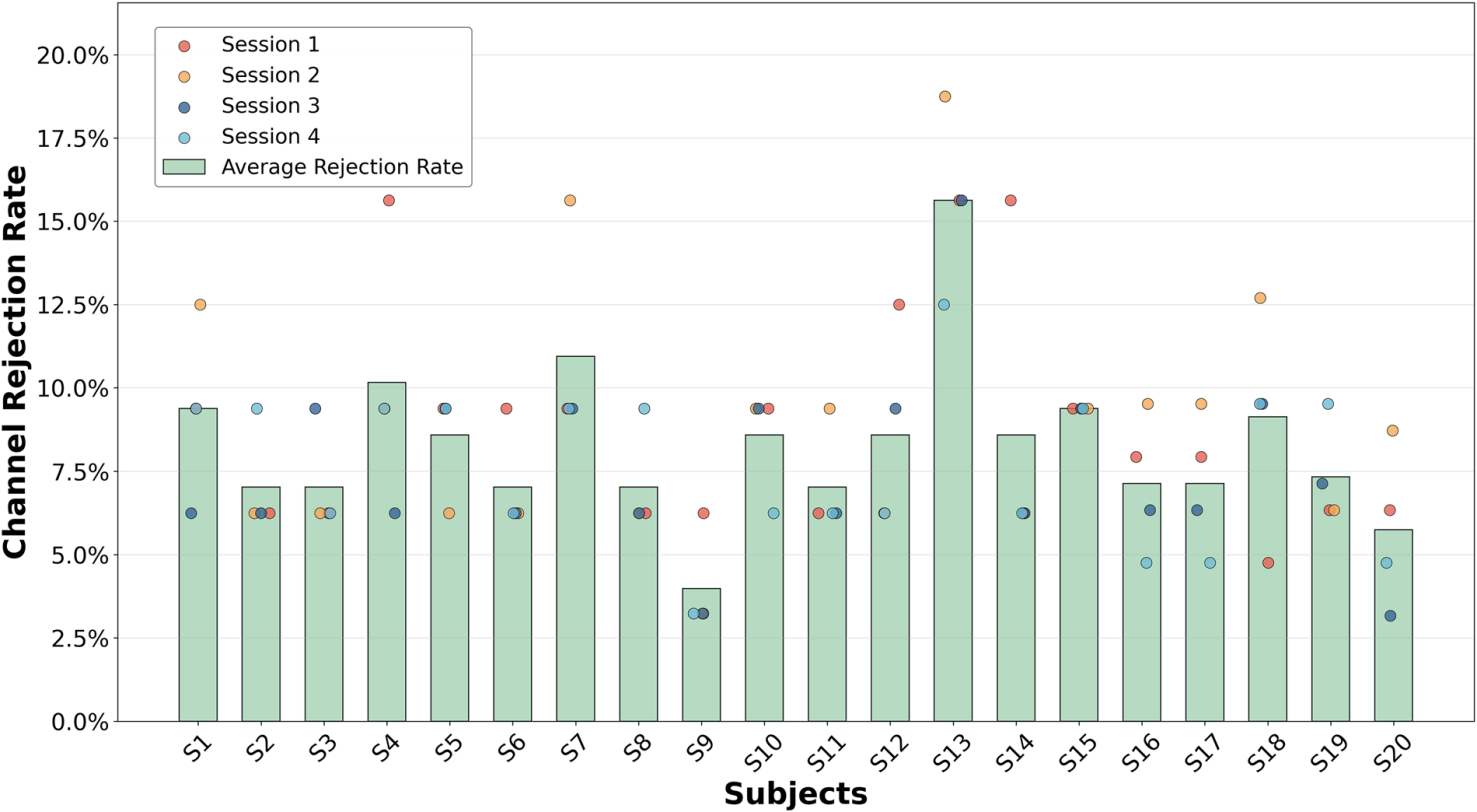
Channel Rejection Rate per Session and the Average across All Participants.

The channel rejection rate refers to the proportion of channels permanently excluded from subsequent analysis due to unacceptably low signal quality during data preprocessing. This metric directly reflects the channel-level quality of an individual subject’s EEG data. A high rejection rate typically indicates potential issues such as hardware connection faults, excessive head movement by the subject, or severe contamination by physiological artifacts. It should be noted that this study predefined the removal of two EOG channels associated with eye-movement artifacts for each subject. Under this premise, a calculated channel rejection rate between 6.25% and 12.5% is considered low, indicating good signal quality for that subject. As shown in Fig. 5, the average trial-wise channel rejection rate across all trials, as well as the rejection rates for individual trials, were excellent for all subjects except S13. This overall demonstrates the high quality of the data collected in this experiment.

## EEG frequency domain feature analysis based on PSD estimation

**Fig. 6:**
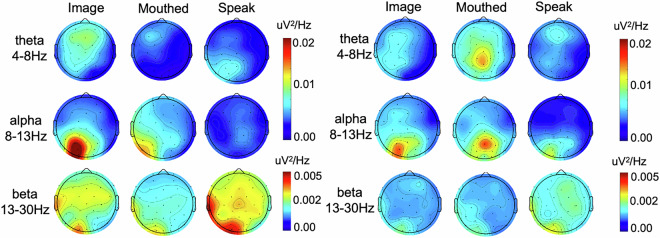
Spectral Analysis of EEG Features. Topographic maps depict Power Spectral Density (PSD) distributions across individual subjects: (**a**) Subject 1, (**b**) Subject 2. Features are displayed separately for each frequency band.

Distinct neural oscillations mediate specialized information processing mechanisms. For instance, theta oscillations (4–8 Hz) relate to working memory and cognitive control, while alpha rhythms (8–13 Hz) reflect attentional and inhibitory processes. In EEG spectral analysis, power spectral density (PSD) serves as a key metric quantifying neural signal power distribution within defined frequency bands. To investigate these functionally specific neural activities and compare band-specific activation patterns across brain regions, we computed PSD values for theta, alpha, and beta bands at each electrode using preprocessed data. We implemented Welch’s method to calculate PSD,

1 $${{\rm{P}}}_{{\rm{l}}}({\rm{W}})=\frac{1}{{\rm{n}}}{\left|\mathop{\sum }\limits_{{\rm{m}}=0}^{{\rm{n}}-1}{\rm{\varepsilon }}({\rm{m}}){{\rm{e}}}^{-{\rm{jwm}}}\right|}^{2}$$

where j denotes the imaginary unit, c indexes EEG channels, W represents frequency, n indicates data points per channel, N is total acquired signals, and x_c corresponds to EEG signals from channel c. Equation 1 derives channel-specific PSD across frequency bands. Figure 6 visualizes topographical maps of theta, alpha, and beta bands during three speech states for participants Sub1 and Sub2. In Sub1, overt speech exhibited maximal theta power, whereas alpha dominated during silently articulated and imagined speech. Topographically, imagined speech showed strongest theta in prefrontal/central regions. Peak alpha during imagined speech localized to parietal areas, indicating an internalized state requiring external interference suppression and focused mental imagery. Maximal beta power during overt speech occurred in central/parietal regions, with imagined speech exceeding silently articulated levels. Sub2 demonstrated predominant alpha power across all speech states. Theta peaked during silently articulated speech over central/parietal cortex. Elevated alpha during imagined and silently articulated speech implies active suppression of somatosensory feedback to maintain mental simulation or inhibit vocalization. Left-central beta enhancement during overt speech, with minimal activity in other states.

## Spatial Feature Analysis Based on Common Spatial Patterns

**Fig. 7:**
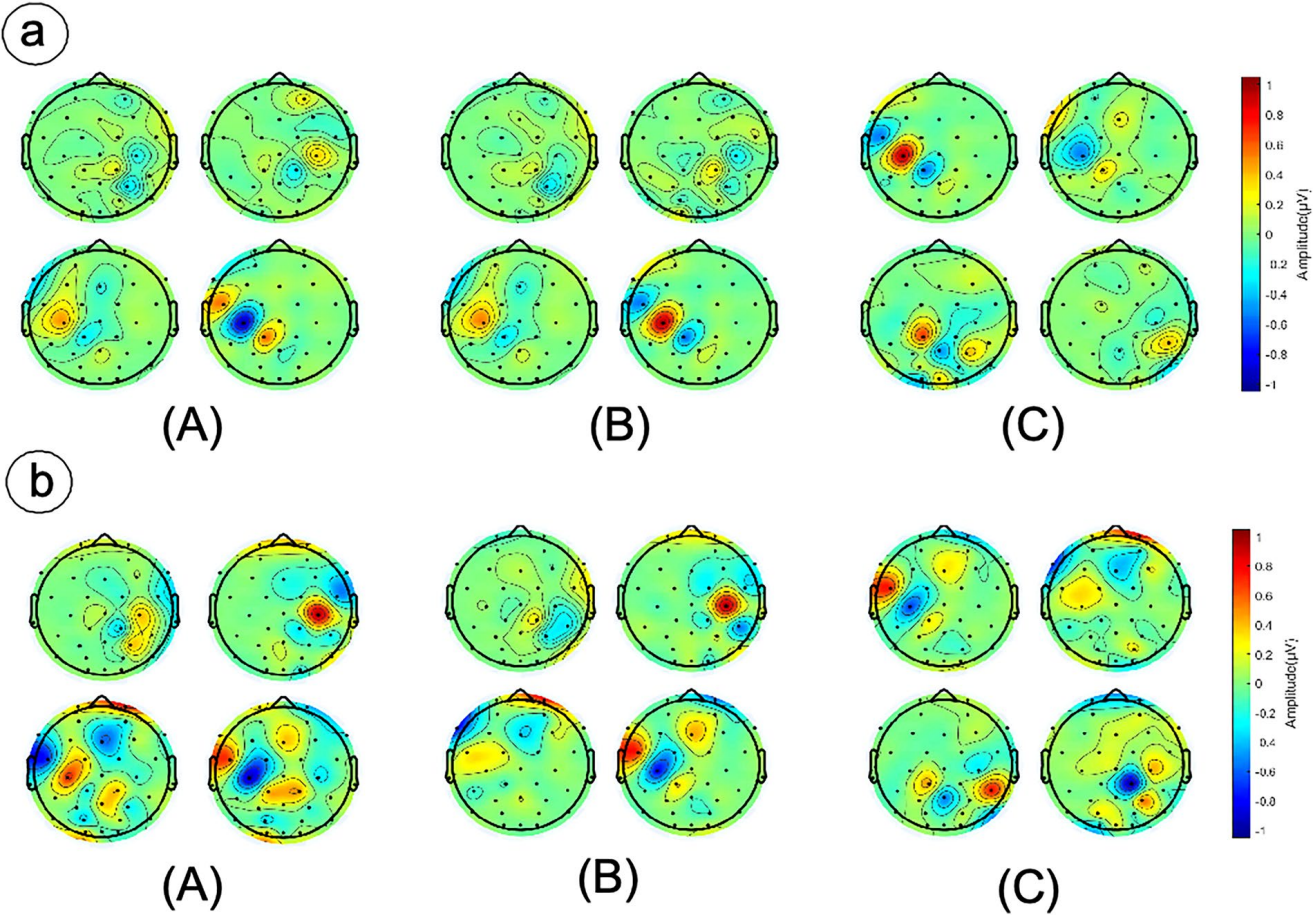
Spatial Domain Feature Analysis of EEG. Common Spatial Pattern (CSP) derived topographies showing the 4 most significant spatial patterns for two subjects: (**a**) Subject 1, (**b**) Subject 2. Panels (A)-(C) respectively contrast task stages: ‘Speak vs. Silently articulated’, ‘Speak vs. Imagine’, and ‘Imagine vs. Silently articulated’. Each subplot displays the 4 dominant spatial patterns.

To characterize spatial activation patterns across speech modalities and localize cortical regions for linguistic functions, we performed CSP-based spatial analysis. As a supervised spatial filtering method^44^, CSP’s core strength lies in maximizing discriminative spatial features between two EEG signal classes. The algorithm derives spatial filters through mathematical optimization that maximize variance for one signal class while minimizing variance for the other. Spatial filter weights directly map to scalp electrode contributions, enabling neuroscientifically interpretable results. Figure 7 displays CSP topographic maps contrasting three speech states for Sub1 and Sub2 using preprocessed data.

Inter-subject CSP variations suggest distinct internal simulation strategies during imagined speech. For Sub1, the overt vs silently articulated contrast revealed dominant activation near the central sulcus during overt speech—a region governing motor execution—while silently articulated speech engaged prefrontal cortex and bilateral supplementary motor area (SMA), consistent with cognitive control and internal modeling. In the overt vs imagined contrast, imagined speech showed pronounced primary motor cortex (M1) activation compared to overt speech, indicating articulatory imagery (e.g., lip/tongue positioning) predominantly controlled by M1 rather than language regions. During the imagined vs silently articulated comparison, imagined speech maintained this M1 dominance, though weaker motor cortex activation was also observable during silently articulated speech. This pattern confirms Sub1’s motor-centric simulation strategy across all speech states. Conversely, Sub2 (Fig. 7b) exhibited fundamentally different activation patterns. The overt vs silently articulated contrast highlighted central sulcus activation during overt speech, reflecting complex articulatory control, while silently articulated speech recruited dorsolateral prefrontal cortex (DLPFC) and anterior cingulate cortex (ACC), implicating motor suppression and working memory maintenance. Notably, in the imagined vs silently articulated comparison, imagined speech demonstrated striking activation in Broca’s area, suggesting a phonological simulation strategy where participants internally rehearsed pinyin sounds, thereby engaging core language production regions rather than motor execution circuits.

### EEG source reconstruction

We analyzed imagined articulation data from both experimental groups using MNE-Python. Cortical surface reconstruction was performed with MNE’s fsaverage MRI template^45^ (https://surfer.nmr.mgh.harvard.edu/fswiki/FsAverage). A three-layer Boundary Element Method (BEM) model was created with 15,360 triangular facets, with conductivities set at 0.3 S/m (brain), 0.006 S/m (skull), and 0.3 S/m (scalp). The source space comprised 1,281 sources per hemisphere (total 2,562 sources). Preprocessed data from the imagined articulation phase (bandpass-filtered at 4–45 Hz) served as input, and the electrode positions were defined according to the standard 10–05 (1005) montage template. The inverse solution was computed using the widely adopted dynamic Statistical Parametric Mapping (dSPM) approach^46^.

**Fig. 8:**
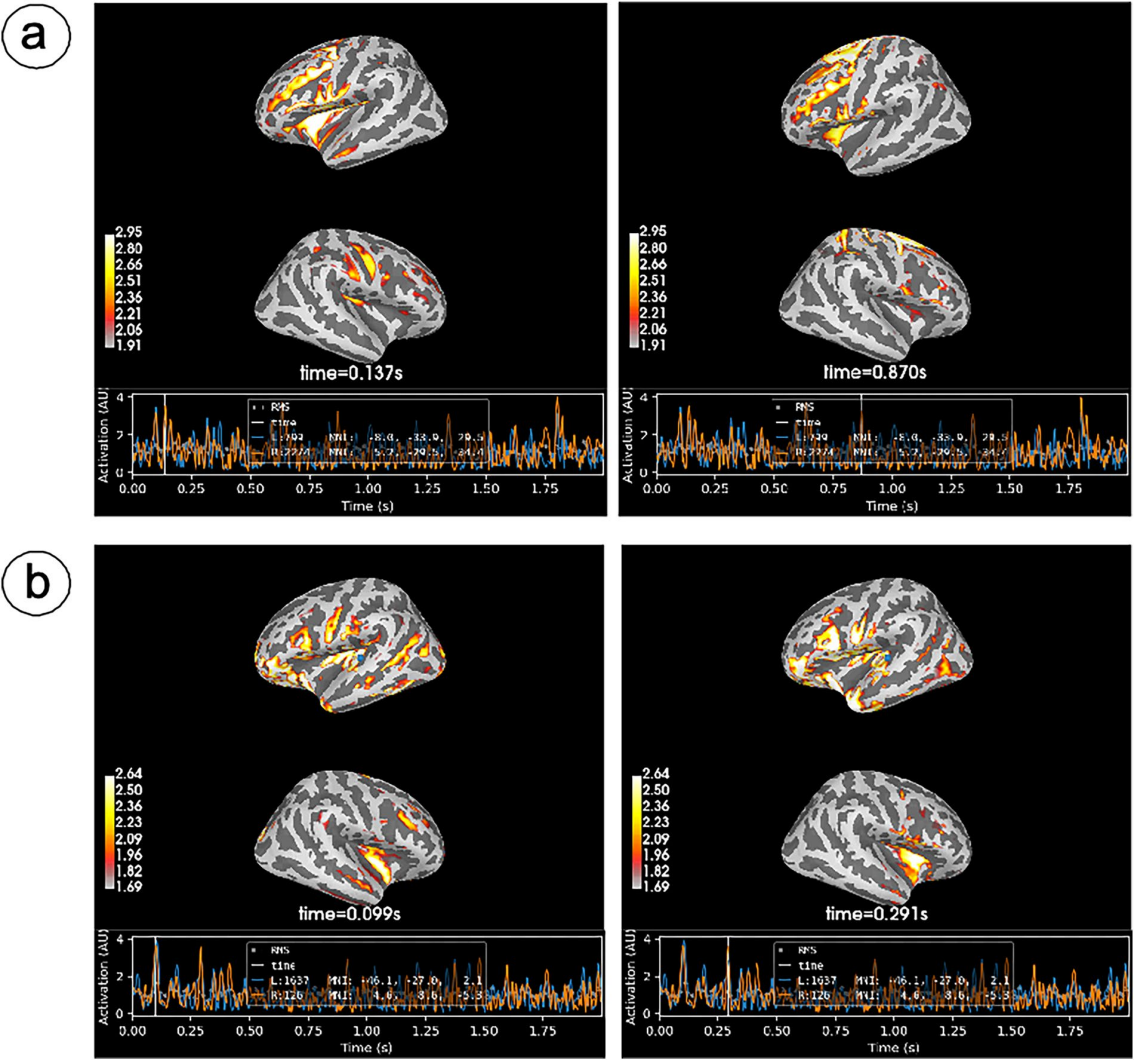
EEG source localization analysis. Cortical activation maps derived from source reconstruction for two subjects: (**a**) Subject 1, (**b**) Subject 2. The dynamic Statistical Parametric Mapping (dSPM) approach was employed to solve the inverse problem. Within each subplot, the upper panel represents left-hemisphere activation while the lower panel displays right-hemisphere activation. Visualization corresponds to peak activation time points within selected hemispheric regions.

Source localization results are visualized in Fig. 8 with separate displays for left and right hemispheres, capturing peak activation moments. Building on CSP findings, we further analyzed these two phenotypically distinct participants. Sub1 exhibited concentrated activation in: (1) the sensorimotor cortex near the central sulcus, likely reflecting articulatory motor memory during overt speech recall; (2) the inferior temporal gyrus encompassing Wernicke’s area (auditory language processing); and (3) the supplementary motor area (SMA) in the superior medial frontal lobe. Compared to Sub2’s activation pattern, Sub1 showed stronger engagement of motor regions (sensorimotor cortex and SMA) but weaker Broca’s area involvement. Notably, both participants demonstrated significant Wernicke’s area activation. Conversely, Sub2’s activation primarily clustered in: (1) Broca’s area within the inferior frontal gyrus, which coordinates speech motor planning and production^47^; and (2) Wernicke’s area in the posterior superior temporal gyrus, supporting language comprehension. This pattern suggests imagined speech activated phonological/auditory representations and semantic associations specific to each pinyin stimulus^48^.

### Classification validation using baseline models

Beyond neural activity analysis, we leveraged deep learning models to perform feature extraction and decoding on the collected EEG signals. EEGNet is widely adopted for EEG decoding^49^, serving as our baseline model for classification, using 10-fold cross-validation to calculate accuracy. Proposed by Lawhern *et al*., this compact convolutional neural network is specifically designed for EEG classification, employing parameter sharing and depthwise separable convolutions to reduce complexity, making it suitable for limited EEG datasets^50^.

**Fig. 9:**
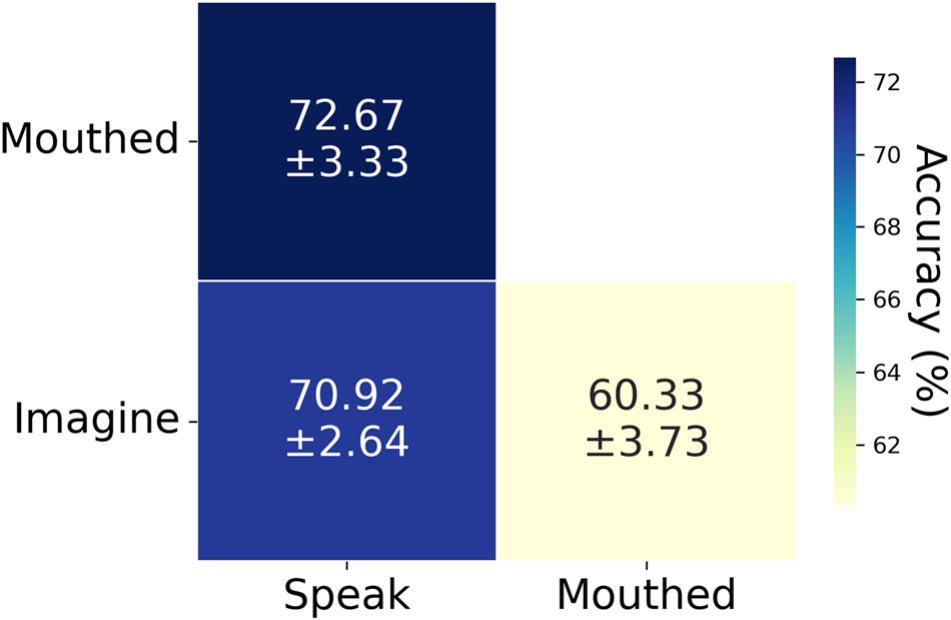
Binary classification accuracy across three speech production modes.

**Fig. 10:**
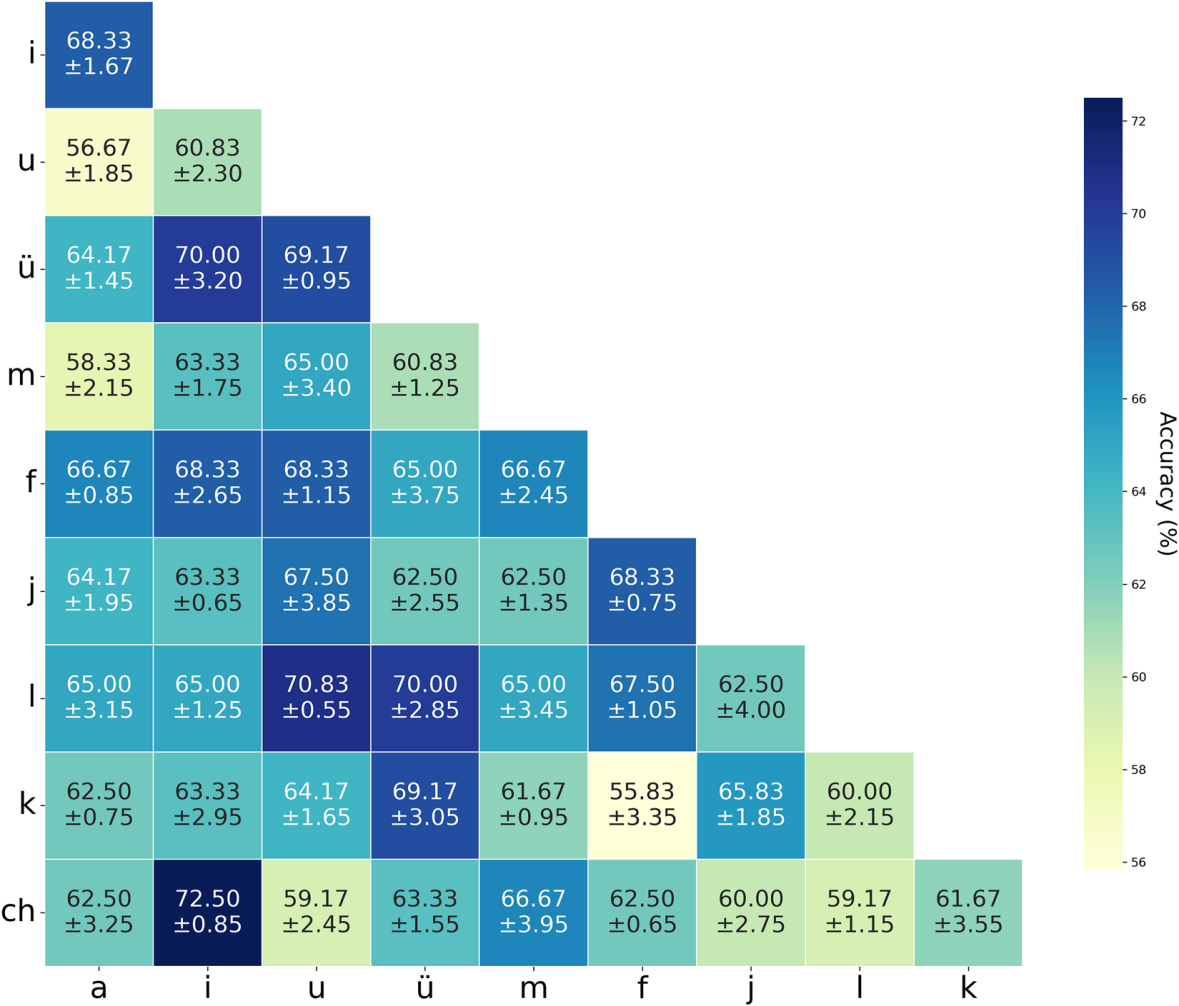
Binary classification accuracy for different Pinyin syllables during imagined speech trials.

Figure 9 presents classification results across articulation modes (Subject 1): Speak vs. Silently articulated: 72.67 ± 3.33%; Speak vs. Imagine: 70.92 ± 2.64%. Silently articulated vs. Imagined: 60.33 ± 3.73%. All three articulation modes were distinguishable, with performance marginally surpassing prior studies^51^. Figure 10 shows pairwise classification of Pinyin syllables during imagined articulation (Subject1), with all accuracies >55% (peak: 72.5 ± 0.85%).

## Usage Notes

All experimental and processing codes are available on GitHub for public access. Code was developed using Python 3.12 and MATLAB® 2024. MATLAB® 2024 with EEGLAB was primarily used for data preprocessing, while Python 3.12 with MNE-Python v1.6.0 implemented validation pipelines. EEG preprocessing is provided as step-by-step documentation, enabling parameter customization (downsampling, filter bands, ICA denoising). Researchers can adapt pipelines for specific requirements. Stimulus presentation was implemented in Python.

## Data Availability

In line with the principles of reproducible research, the dataset associated with this article—including participants’ basic demographic information (age, gender, handedness), detailed acquisition device parameters, event information, raw data, and preprocessed data—are freely available on the OpenNeuro platform (https://openneuro.org/datasets/ds006465/versions/2.0.0)^43^. Public data is distributed under the Creative Commons Attribution 4.0 International Public License (https://creativecommons.org/licenses/by/4.0/). Researchers may use the raw data to perform additional processing tailored to their specific experimental needs, or directly utilize the preprocessed data we have provided.

## Code availability

In line with the philosophy of reproducible research, all codes used in this paper, including those for stimulus presentation, data preprocessing, and technical validation, are accessible at Github (https://github.com/XinyuM-OvO/3M-CPSEED). Users can customize parameters to meet specific research requirements.
